# Two-Year Outcome of Synthetic Annular Ligament Reconstruction in the Elbow: A Case Report

**DOI:** 10.7759/cureus.11564

**Published:** 2020-11-19

**Authors:** Levin Kesu Belani, Shalimar Abdullah, Mohd Hezery Harun, Parminder Singh Gill Narin Singh, Jamari Sapuan

**Affiliations:** 1 Orthopaedic and Traumatology, Faculty of Medicine, Universiti Kebangsaan Malaysia, Kuala Lumpur, MYS; 2 Orthopaedic and Traumatology, Universiti Putra Malaysia, Kuala Lumpur, MYS

**Keywords:** monteggia's fracture, fracture dislocation, suture anchors, annular ligament, elbow, radial head instability, annular ligament reconstruction

## Abstract

Monteggia fracture is commonly treated with open anatomical reduction and fixation of the ulna fracture. The radial head will be automatically reduced once anatomical fixation of the ulna is achieved. However, it is occasionally associated with an irreducible radial head dislocation requiring an open reduction and reconstruction of the torn annular ligament. We describe a case of traumatic Monteggia fracture which underwent initial plating, however post-operative radiograph denoted an irreducible radial head secondary to a ruptured annular ligament. We reconstructed the annular ligament with a synthetic graft sling around the radial neck with an anchor suture. The radial head was stable in all directions after annular ligament reconstruction. A two-year follow-up shows full range of motion of the elbow joint with osteolysis of the radial head, no other operative morbidity was observed.

## Introduction

Monteggia fracture is characterized by fracture of the ulna shaft with dislocation of the radial head. Bado had classified Monteggia fracture into four types based on the direction of radial head dislocation and displacement of ulna fracture [1]. The aim of management of Monteggia fracture is early detection of injury, followed by anatomical reduction of ulna and radial head. Chronic radial head dislocation may require additional procedures such as ulnar and radial osteotomies, annular ligament repair or reconstruction and transarticular temporary wire to fix the radial head [2].

The annular ligament is a strong band of fibers encircling the head of the radius, and retains it in contact with the radial notch of the ulna. It prevents dislocation by limiting forward, backward and lateral displacement of the radial head [3]. Galik et al. have identified that the annular ligament is important in preventing anteroposterior and mediolateral translation of the radial head [4].

There are several techniques of annular ligament reconstruction for treatment of radial head dislocation [2-3,5-9]. These techniques vary in terms of source of graft and methods of fixation. There are disadvantages of autografts such as donor site morbidity, large or additional incision for graft harvesting, and the strength and size of the graft can be unpredictable. Furthermore, the duration of surgery will be lengthened by the additional procedures.

Wang et al. utilized palmaris longus grafts with suture anchors in 20 children with radial head dislocations with successful results [5]. Rather than utilizing autografts with its associated complications, we propose annular ligament reconstruction using a synthetic ligament slung around the radial head and fixed to bone with anchor suture. The synthetic ligament is a polyethylene terephthalate (polyester) (Neoligaments, Leeds, UK) which is non-absorbable, and provides high strength and stiffness. We intend to highlight the patient functional and surgical outcome of reconstructing the annular ligament using a synthetic ligament.

## Case presentation

A 45-year-old right-hand-dominant healthy male was thrown off his motorcycle and landed on his right upper limb. He presented with pain and deformity of his right forearm. His right elbow and forearm were swollen, deformed and tender along the ulna border. He was unable to actively move his right elbow due to pain.

**Figure 1:**
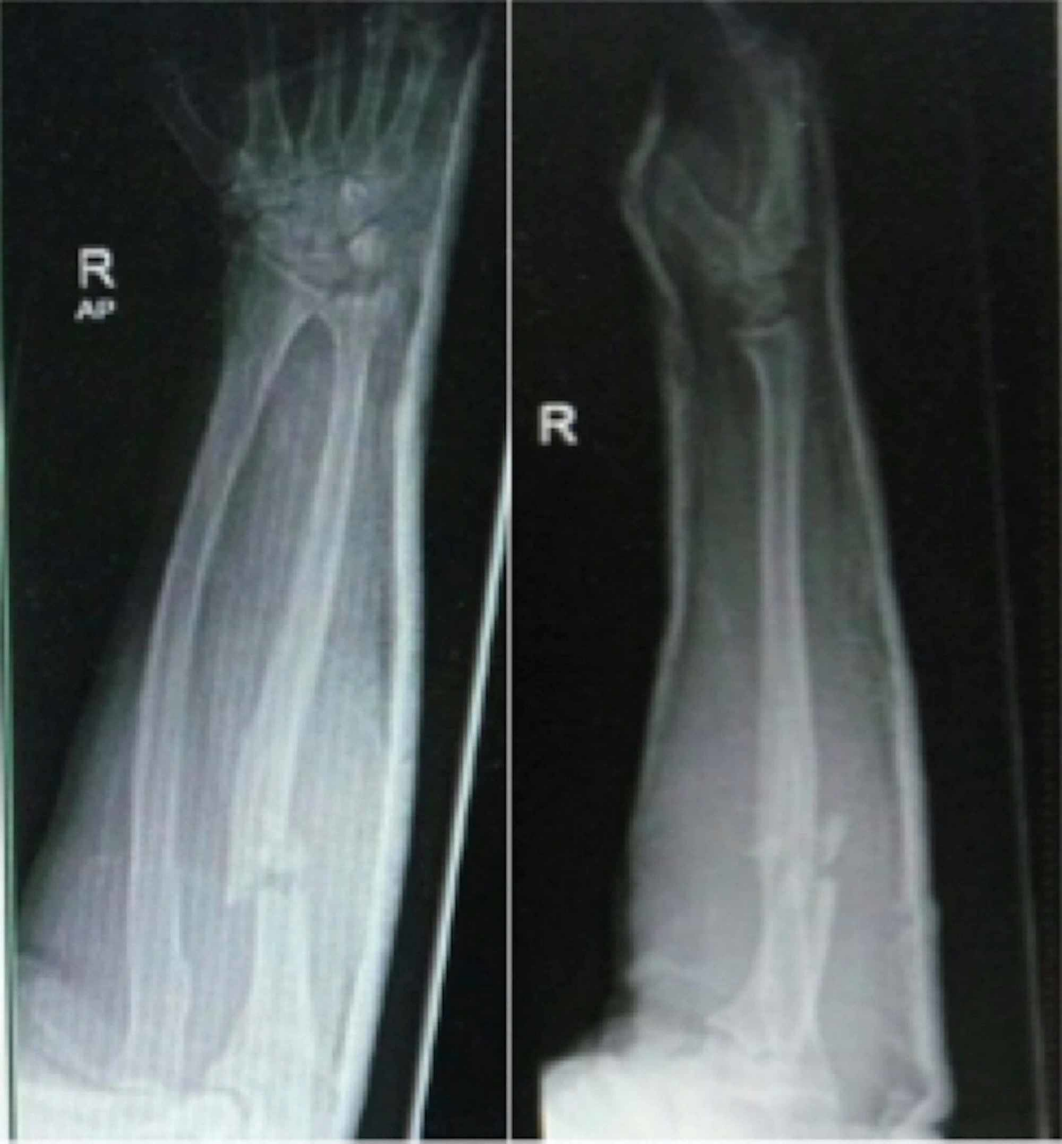
Radiograph showing Monteggia fracture of right forearm (AP and lateral view).

**Figure 2:**
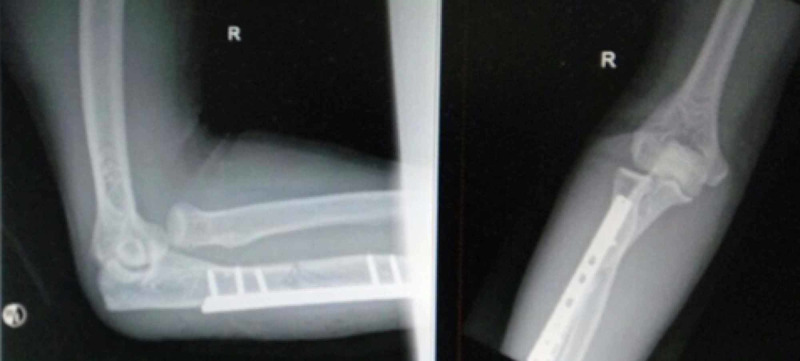
Radiograph showing irreducible radial head dislocation after fixation of ulna fracture (AP and lateral view).

Radiographs of right forearm and elbow joint revealed a Monteggia fracture, Bado class I (anterior) (Figure 1). Anatomical reduction and stable fixation of ulna fracture with dynamic compression plate was achieved during surgery. However, during post-operative check radiograph, there was a persistent radio-capitellar dislocation with an anterior displacement of the radial head (Figure 2). An ultrasound of the elbow confirmed annular ligament tear. A second stage surgery to reduce and reconstruct the annular ligament by using synthetic ligament was done three days later.

The patient was placed in supine position, general anaesthesia administered, prophylactic antibiotic (IV cefuroxime 1.5 g) given at induction and tourniquet was applied on the right arm. The right elbow was in flexion and the forearm in pronation to ensure the radial head is prominent. The radio-capitellar joint and radial head was exposed through the Kocher’s approach.

**Figure 3:**
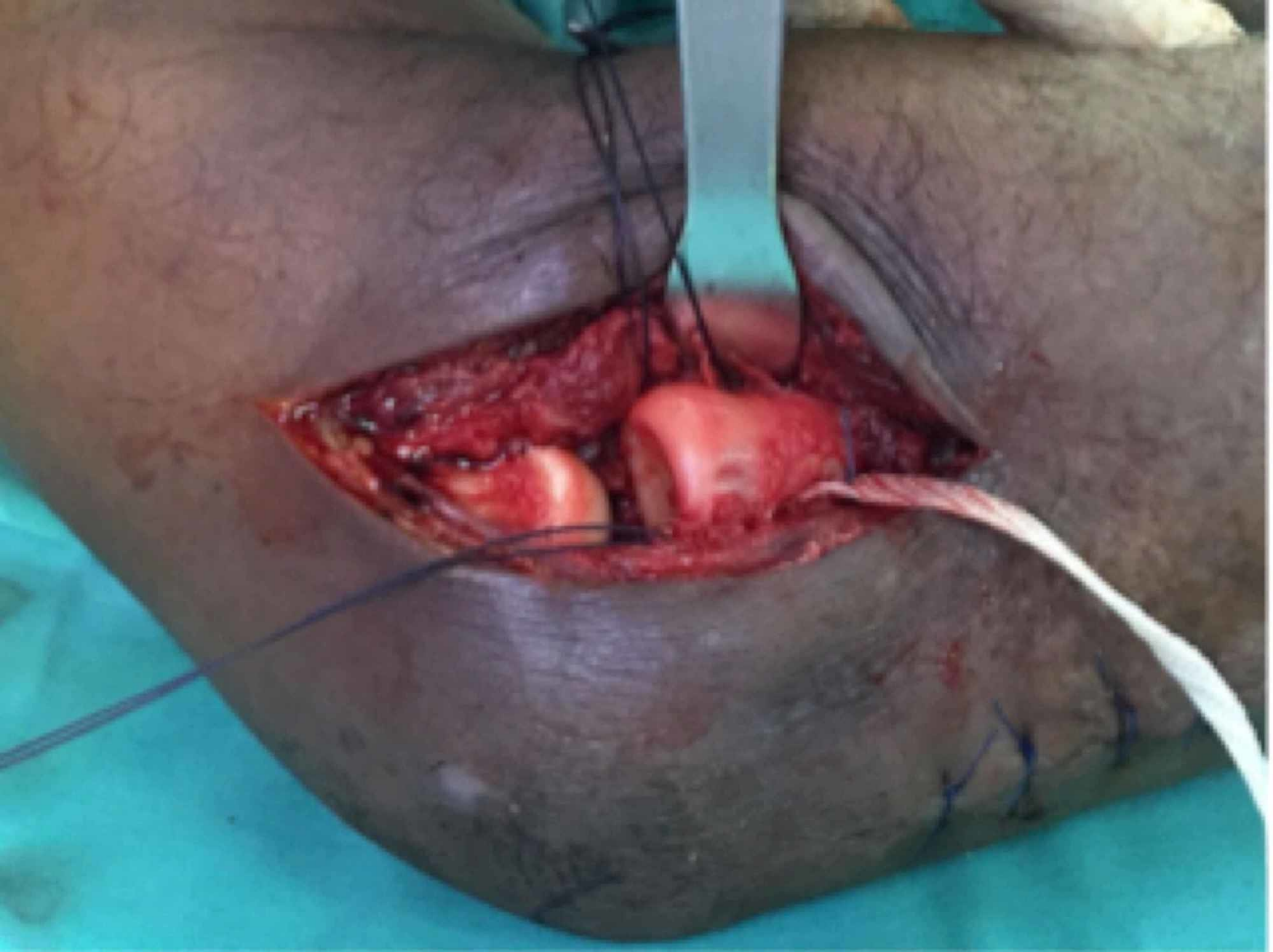
Complex tear of annular ligament. Absorbable sutures (Vicryl 2/0) utilised to introduce synthetic ligament insertion.

**Figure 4:**
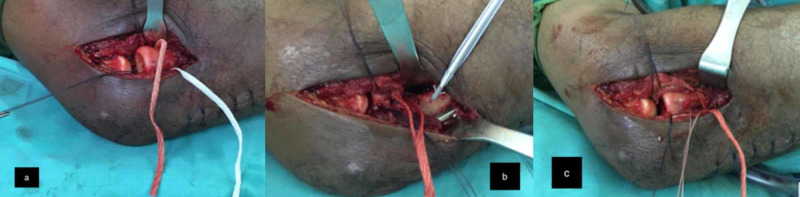
a: Synthetic ligament sling around radial neck. b: Synthetic ligament is tightened before fixation. c: Synthetic ligament fixed to the lateral border of proximal ulna using an anchor suture.

Intraoperatively, the radial head was dislocated anteriorly. The annular ligament was torn and ruptured (Figure 3). The ligament edges were irregular and interposed in the radio-capitellar and proximal radio-ulna joint. Reduction of the radial head was achieved after interposed soft tissues were cleared but was unstable. We proceeded to reconstruct the annular ligament using a synthetic graft. After reduction of the radial head, the synthetic ligament was slung in a simple U-shape configuration around the radial neck and the ends of the ligament anchored by using the anchor suture (Mitek bone anchor, J&J ®) to the lateral border of the proximal ulnar bone (Figure 4a-4c). The force of the anchoring must be just adequate to overcome the anterior deforming force of the radial head, but not too tight that it limits supination and pronation of the forearm. The range of motion (ROM) of elbow and forearm was almost full after reconstruction of the annular ligament. Four centimeters of synthetic ligament and one anchor suture was used.

**Figure 5:**
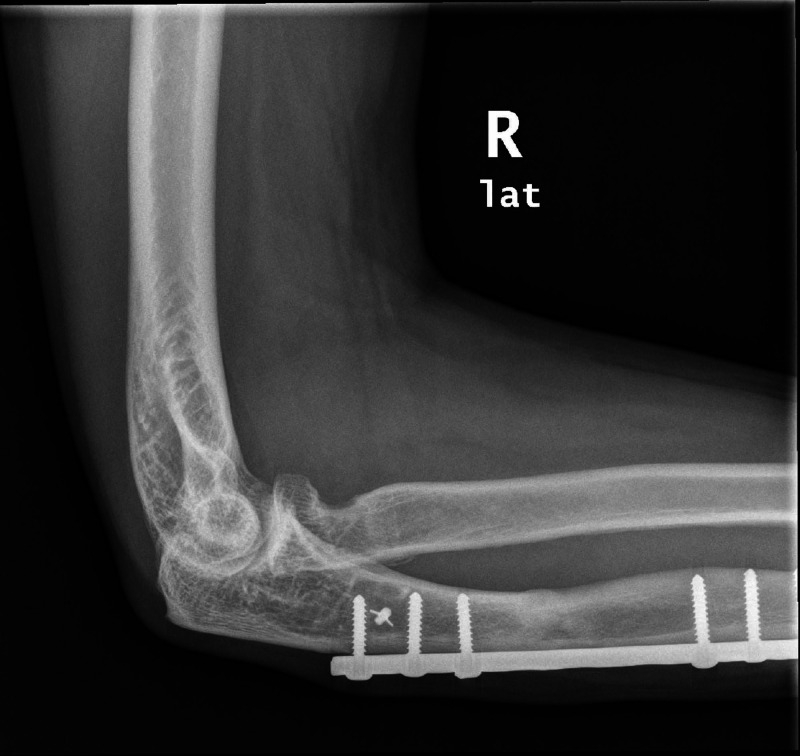
Lateral elbow radiograph two years post-annular ligament reconstruction with osteolysis of the radial head.

Additional stability of the reconstruction was achieved by capsule and superficial fascia repair. The patient was placed in an above elbow splint for one month before allowing full ROM and rehabilitation. At two months post-surgery, his elbow range of motion (flexion) was 0 to 110°, pronation up to 45° and supination up to 60°. A two-year follow-up showed that his elbow range of motion (flexion) was 0-130°, pronation and supination 90°. He is able to play badminton with no limitation of movement or pain. Elbow radiograph at two years post operative shows that the reduction of the radial head is maintained, but with osteolysis of the radial neck (Figure 5).

## Discussion

Traumatic Monteggia fracture with irreducible radial head dislocation after plating of the ulna should raise a further concern. The radial head should align with the capitellum once the ulna is reduced, however, if it does not, the usual cause is misalignment of the ulnar bone. Uncommonly, a soft tissue block may be present and this is most commonly secondary to a ruptured annular ligament. In cases of neglected Monteggia, it would be advisable to prepare for synthetic grafting as well in a one stage procedure.

The annular ligament is attached by both its ends to the anterior and posterior margins of the radial notch of the ulna, together with which it forms the articular surface that surrounds the head and neck of the radius. The ligament is strong and well defined, yet its flexibility permits the slightly oval head of the radius to rotate freely during pronation and supination. By virtue of the radial head being wider than the radial neck and since the annular ligament embraces both, the radial head is “trapped” inside the ligament preventing distal displacement of the radius [10]. Given that the annular ligament gives both transverse and longitudinal plane stability, joint preserving annular ligament reconstruction is indicated in cases of proximal radio-ulnar joint instability such as the case described above [11]. The indication of annular ligament reconstruction is well understood, although there are no conclusive evidence on a particular surgical technique [12].

Bell Tawse in 1965 first described open reduction and annular ligament reconstruction with a slip of triceps tendon for malunited Monteggia fracture [6]. Cappellino et al. retrospectively reviewed five patients with chronic acquired radial head dislocation treated with modified Bell Tawse procedure [7]. They concluded that reconstruction of annular ligament using a strip of triceps tendon is an effective treatment with good functional outcome [7].

Boyd and Boals recommended open reduction and plating of ulna for adult Monteggia fracture [8]. In majority of cases the radial head will be reduced spontaneously after fixation of ulna. If the reduction is unstable, removal of fibrous tissue in the joint is recommended and the annular ligament is repaired. Fascial loop technique is employed if primary repair of annular ligament is not feasible [8].

Annular ligament reconstruction with suture anchor is another technique to treat radial head dislocation. The suture anchor is a bioabsorbable implant and a great fixation device for reattaching tendons and ligaments to bone i.e. palmaris longus tendon autograft with suture anchor is an excellent treatment for radial head dislocation with full functional outcome in children [2]. However, this technique is associated with complications such as stiffness, bone nonunion or malunion, avascular necrosis, and pain [2]. Other method of fixation of tendon autograft was described by Tan et al. whereby the triceps tendon strip is passed through two ulna bone tunnel and tied with a suture [9]. Based on an anatomic study by Nwoko et al., the tendon of the superficial head of brachialis muscle can be used as a distally based tendon graft for annular ligament reconstruction [3].

The methods above describe annular ligament reconstructions with autograft and anchor sutures. We describe a method of annular ligament reconstruction using a synthetic ligament slung around the radial head and fixed to bone with anchor suture. The synthetic ligament is a polyethylene terephthalate (polyester) (Neoligaments, Leeds, UK), better known as Leeds-Keio ligament, which is non-absorbable, and provides high strength and stiffness. It is used for tissue approximation, tendon and ligaments reconstruction and as substitute for tendon autografts in patients with massive tendon loss [13,14]. This method was chosen to prevent utilization of a donor autograph leading to an increased morbidity. The Leeds-Keio ligament has been utilized in multiple reconstruction surgeries for the Anterior Cruciate Ligament, however no literature has reported its use in the elbow as far as we know. We can see early bony erosion occurring at the radial neck of our patient (Figure 5). This erosion appears similar to those following acromioclavicular joint reconstruction utilising synthetic ligaments [15,16].

## Conclusions

Besides providing stable and adequate construct and fixation, our approach of annular ligament reconstruction using synthetic ligament minimizes donor site morbidity, shortens surgical time and surgical incision encouraging better wound recovery. Range of motion of elbow and forearm was good post-operatively with stable elbow joint despite the osteolysis observed.
